# Chronic Presence of Oligomeric Aβ Differentially Modulates Spine Parameters in the Hippocampus and Cortex of Mice With Low APP Transgene Expression

**DOI:** 10.3389/fnsyn.2020.00016

**Published:** 2020-04-24

**Authors:** Mariya V. Hrynchak, Marina Rierola, Nataliya Golovyashkina, Lorène Penazzi, Wiebke C. Pump, Bastian David, Frederik Sündermann, Roland Brandt, Lidia Bakota

**Affiliations:** ^1^Department of Neurobiology, School of Biology/Chemistry, University of Osnabrück, Osnabrück, Germany; ^2^Center for Cellular Nanoanalytics, University of Osnabrück, Osnabrück, Germany; ^3^Institute of Cognitive Science, University of Osnabrück, Osnabrück, Germany

**Keywords:** Aβ, Alzheimer’s disease, cortex, dendritic spine, hippocampus

## Abstract

Alzheimer’s disease is regarded as a synaptopathy with a long presymptomatic phase. Soluble, oligomeric amyloid-β (Aβ) is thought to play a causative role in this disease, which eventually leads to cognitive decline. However, most animal studies have employed mice expressing high levels of the Aβ precursor protein (APP) transgene to drive pathology. Here, to understand how the principal neurons in different brain regions cope with moderate, chronically present levels of Aβ, we employed transgenic mice expressing equal levels of mouse and human APP carrying a combination of three familial AD (FAD)-linked mutations (Swedish, Dutch, and London), that develop plaques only in old age. We analyzed dendritic spine parameters in hippocampal and cortical brain regions after targeted expression of EGFP to allow high-resolution imaging, followed by algorithm-based evaluation of mice of both sexes from adolescence to old age. We report that Aβ species gradually accumulated throughout the life of APP_SDL_ mice, but not the oligomeric forms, and that the amount of membrane-associated oligomers decreased at the onset of plaque formation. We observed an age-dependent loss of thin spines under most conditions as an indicator of a loss of synaptic plasticity in older mice. We further found that hippocampal pyramidal neurons respond to increased Aβ levels by lowering spine density and shifting spine morphology, which reached significance in the CA1 subfield. In contrast, the spine density in cortical pyramidal neurons of APP_SDL_ mice was unchanged. We also observed an increase in the protein levels of PSD-95 and Arc in the hippocampus and cortex, respectively. Our data demonstrated that increased concentrations of Aβ have diverse effects on dendritic spines in the brain and suggest that hippocampal and cortical neurons have different adaptive and compensatory capacity during their lifetime. Our data also indicated that spine morphology differs between sexes in a region-specific manner.

## Introduction

Alzheimer’s disease (AD) is the most common form of dementia in the elderly. The key histopathological features of AD are the formation of plaques consisting of extracellular deposits of β-amyloid (Aβ) peptides and intraneuronal neurofibrillary tangles (NFTs) composed of a hyperphosphorylated form of the microtubule-associated protein tau.

According to the amyloid hypothesis, Aβ deposits are thought to play a causative role in AD (Hardy and Selkoe, 2002), suggesting that Aβ accumulation initiates a cascade of events that result in synaptic changes, tau pathology, and neuron loss that eventually leads to cognitive decline (Bakota and Brandt, 2016). Although the amyloid hypothesis was initially based on the fact that insoluble Aβ aggregates could be observed in the brains of AD patients, it has changed to reflect the growing evidence that soluble, oligomeric forms of Aβ may represent the major neurotoxic species (Lacor et al., 2007; Shankar et al., 2008; Tu et al., 2014). Missense mutations in the genes coding for amyloid beta precursor protein (APP) or APP processing enzymes such as presenilin (PSEN) in familial forms of AD (FAD) have been found to result in increased amounts of Aβ. This has led to the generation of several mouse models transgenic for human *APP* or other disease-relevant genes harboring FAD-related mutations (Games et al., 1995; Moechars et al., 1999; Richardson and Burns, 2002; Jankowsky and Zheng, 2017). Recently, spontaneous cases of AD have also been identified where patients contained more DNA and increased *APP* copy number (Bushman et al., 2015), likely due to somatic gene recombination (Lee et al., 2018). This confirmed an important role in the long-term increase in Aβ production in the development of the disease. AD is considered a disease of synaptic failure (Selkoe, 2002; Arendt, 2009), which occurs substantially earlier than intense neuronal degeneration and plaque formation. The existence of a long presymptomatic/preclinical phase of AD indicates that the chronic exposure to low or moderate amounts of soluble Aβ might induce subtle changes in synaptic connectivity long before the emergence of cognitive impairment. It is also conceivable that AD could be treated before cognitive deficits and massive neurodegeneration occur to delay the onset of clinical symptoms. Consequently, it has recently become apparent that models of aging are needed to investigate age-related neurodegeneration (Johnson, 2015). Moreover, several studies have proposed the use of knock-in mice or mice with low overexpression of the transgene to better mimic the long-term progression of the disease (Saito et al., 2016). In such a context, transgenic mice that express moderate concentrations of soluble Aβ in a sustained manner and develop plaques only late in life could be instrumental for analyzing presymptomatic and chronic Aβ effects during aging. Mice transgenic for human APP695 with the combination of Swedish (KM595/596NL), Dutch (E618Q), and London (V642I) mutations under the control of the platelet-derived growth factor beta (*PDGFB*) promoter (APP_SDL_ mice) produce moderate levels of Aβ and develop plaques only in old age (Blanchard et al., 2003). These mice have proven to also be useful for the generation of *ex vivo* models since they already express Aβ40 and Aβ42 at early postnatal stages (Tackenberg and Brandt, 2009; Golovyashkina et al., 2015; Penazzi et al., 2016). It has been shown that male APP_SDL_ mice have a reduced olfactory habituation and a higher level of anxiety compared to control mice, but have no significant deficits in hippocampus-related spatial memory at 17–18 months when plaque formation starts to emerge (Penazzi et al., 2017). A deficit of olfactory function or anxiety-like behavior has been associated with amyloidosis-related pathologies and can be an informative biomarker for diagnosing the earliest stage of neuropathologies such as AD (Lee et al., 2004; Alvarado-Martínez et al., 2013).

The loss of synapses and dendritic spines, which represent the major excitatory input, is one of the common defects found in human AD brains (for a review see Tackenberg et al., 2009). Spine loss in specific brain regions appears to be an early event during disease development as individuals with mild AD already have fewer synapses (55%) in the *stratum radiatum* of the CA1 subfield (Scheff et al., 2007). A recent detailed 3D ultrastructural analysis performed on the transentorhinal cortex of AD patients showed that a reduction in the percentage of synapses affects the subset of asymmetric synapse types targeting spine heads (Dominguez-Álvaro et al., 2019). Results from human studies also indicate that spines become deformed during AD compared with those in normal aged brains (Baloyannis et al., 2007). This could be functionally relevant because spines with larger heads (mushroom spines) are thought to have stronger synapses (Matsuzaki et al., 2001) and provide higher compartmentalization (Brandt and Paululat, 2013). Aβ induces acute alterations in dendritic spines, as demonstrated in hippocampal cell and tissue culture experiments (Lacor et al., 2007; Shankar et al., 2008; Ortiz-Sanz et al., 2020), and spine loss is also seen in the brains of APP or PSEN transgenic mice (Smith et al., 2009; Merino-Serrais et al., 2011; Zago et al., 2012; Liang et al., 2019). Interestingly, several studies have also shown that short exposure to picomolar concentrations of Aβ positively modulates synaptic plasticity, while prolonged exposure or high (nanomolar) levels of Aβ impair synaptic transmission and induce neuronal loss (Puzzo et al., 2008; Koppensteiner et al., 2016). Therefore, the concentration, type, and duration of Aβ exposure, and likely also the type of receiving neuron, may determine the effect of Aβ on synaptic connectivity.

We hypothesized that the directionality or magnitude of the alterations may differ in various brain regions, depending on the adaptive/compensatory capacity of the respective region. However, to date, relatively few studies have examined early changes in synaptic connectivity before plaque formation, and no study has evaluated the effect of long-term, chronically present soluble Aβ species on changes in spine density and morphology. Furthermore, there are no reliable studies that would provide comparative knowledge of timeline trajectories regarding spine alteration in different brain regions during the long presymptomatic phase characterized by increasing Aβ levels. Finally, to the best of our knowledge, the evolution of dendritic spine parameters in the presence of elevated Aβ levels has not been studied in parallel in both sexes in AD mouse models.

Our motivation was to fill this knowledge gap by analyzing the brain of mice exposed to gradually increasing amounts of soluble Aβ species. We employed a mouse model (APP_SDL_ mice) that produces moderate levels of Aβ peptides throughout life and develops plaques only at an advanced age. To obtain a global understanding of how principal neurons in different brain regions cope with the chronic presence of Aβ from adolescence to old age, we analyzed two hippocampal and two cortical brain regions by algorithm-based evaluation of high-resolution cLSM image stacks. The structures could be analyzed due to the expression of EGFP in subsets of neurons (Feng et al., 2000). Moreover, owing to the emerging awareness of gender bias in AD development (May, 2016), we applied our spine parameter analysis to animals of both sexes.

## Materials and Methods

### Animals

Heterozygous APP_SDL_ transgenic mice expressing human APP_695_ with three FAD-related mutations (Swedish [KM595/596NL], Dutch [E618Q], and London [V642I]) were used (Aventis Pharma; Strasbourg, France). The transgene in these mice is under the control of the *PDGFB* promoter (Blanchard et al., 2003). Age-matched C57BL/6 mice (Charles River Laboratories and Harlan Winkelmann) were employed as control. Genotyping of APP_SDL_ transgenic mice was performed from mouse tail DNA by PCR using the following primers: APP-forward, 5′-GTAGCAGAGGAGGAAGAAGTG-3′ and APP-reverse, 5′-CATGACCTGGGACATTCTC-3′. For analysis of synaptic connectivity and morphology, APP_SDL_ mice were crossed with homozygous EGFP-expressing mice (Thy1-GFP line M; obtained with the permission of Josh Sanes, Harvard University, Cambridge, MA, USA). In these mice, EGFP expression is governed by a neuron-specific *Thy1* promoter element (Feng et al., 2000). Littermates expressing EGFP and non-transgenic for human *APP* served as control. All animals were maintained and euthanized according to the National Institutes of Health guidelines and German animal care regulations.

### Antibodies

The following antibodies were used: monoclonal anti-Aβ antibodies (mouse 4G8; Covance, Munich, Germany), monoclonal anti-neuronal nuclei antibody NeuN (mouse; Chemicon, Temecula, CA, USA), monoclonal anti-actin antibody (mouse; Calbiochem; Darmstadt, Germany), monoclonal anti-APP antibody (22C11; Millipore GmbH Schwalbach/Ts., Germany), monoclonal anti-synaptophysin antibody (mouse; Millipore), PSD-95 (7E3-1B8; mouse; Thermo Fisher Scientific, Rockford, IL, USA), monoclonal anti-Arc antibody (C-7; mouse; Santa Cruz Biotechnology, Dallas, TX, USA) and GAPDH (#AB2302; chicken; Millipore). The following anti-Tau antibodies were used: monoclonal phosphorylation independent Tau-5 (mouse; Labvision, Westinghouse, CA, USA), monoclonal AT8 (mouse; Thermo Fisher Scientific, Rockford, IL, USA), polyclonal pS199 (rabbit; Invitrogen, Carlsbad, CA, USA), and monoclonal PHF1 (mouse; a generous gift from Peter Davies, Albert Einstein College of Medicine, Bronx, NY, USA). As secondary antibodies, cyanine 3 (Cy3)-coupled anti-mouse (Dianova, Hamburg, Germany) and peroxidase-conjugated anti-mouse and anti-rabbit antibodies (Jackson Immuno Research, West Grove, PA, USA) were employed.

### Analyses of Aβ Levels

Animals were euthanized by cervical dislocation. The brains were quickly removed, snap-frozen in liquid nitrogen, and stored at −80°C. Sequential extraction was performed essentially as previously described (Shankar et al., 2009). Briefly, frozen right hemibrains without cerebellum were homogenized in 1.1 ml of ice-cold Tris-buffered saline (TBS) containing a cocktail of protease and phosphates inhibitors (1 mM PMSF, 10 mg/ml each of leupeptin and pepstatin, 1 mM EGTA, 1 mM sodium orthovanadate, 20 mM sodium fluoride, and 1 mM sodium pyrophosphate) with 30 strokes using a mechanical Dounce homogenizer. Homogenates were centrifuged at 175,000× *g* and 4°C for 30 min. The supernatant (designated as TBS extract) was aliquoted and stored at −80°C. To solubilize the membrane-bound Aβ, the TBS-insoluble pellet was homogenized in 1.1 ml of TBS containing 1% Triton X-100 plus inhibitors (TBS-TX) with 30 strokes using a Dounce homogenizer, centrifuged at 175,000× *g* and 4°C for 30 min and the resultant supernatant (designated as TBS-TX extract) was aliquoted and stored at −80°C. TBS and TBS-TX extracts were subjected to an ELISA test using commercially available kits (EZHS40, EZHS42; Millipore GmbH Schwalbach/Ts., Germany; oligomeric Aβ ELISA Kit; Biosensis, Thebarton, Australia). The ELISA for the oligomeric Aβ utilizes the MOAB-2 antibody developed by LaDu and coworkers (Youmans et al., 2012). The authors showed that the MOAB-2 antibody specifically detects Aβ and not the precursor molecule APP. In ELISAs, the oligomeric form of the Aβ peptide (o-Aβ) can be assayed independently of the other forms of the molecule when the MOAB-2 monoclonal antibody is used. Cortices and hippocampi were isolated separately from another set of mice and processed as described above in 5× weight/volume extraction solutions. The samples were tested with ELISA Kit KHB3544 (Thermo Scientific, Schwerte, Germany).

### Fixation and Staining

Mice were anesthetized and perfused transcardially with saline followed by fixation in 4% paraformaldehyde (PFA) in phosphate-buffered saline (PBS). Brains were removed, postfixed overnight at 4°C in 4% PFA/PBS, and stored in PBS at 4°C.

For thioflavin S staining, coronal vibratome sections (50 μm) were mounted on positively charged slides (SuperFrost Plus, Menzel). The sections were stained with 1% thioflavin S for 8 min and then differentiated in two changes of 85% ethanol for 3 min each and two changes of 95% ethanol for 3 min each followed by three washes with ddH_2_O. The sections were mounted with Confocal-Matrix^®^ (Micro-Tech-Lab, Graz, Austria) and a coverslip was applied.

For immunohistochemical staining, free-floating coronal sections (50 μm) were used. The slices were treated with 70% formic acid for 30 min at room temperature and washed with PBS. Then, the slices were permeabilized with 0.4% (v/v) Triton X-100 in PBS for 30 min and washed with 1% (w/v) BSA and 0.1% (v/v) Tween 20 in PBS. The slices were incubated overnight at 4°C with primary antibodies. After washing with PBS, the slices were incubated with a Cy3-conjugated secondary antibody and DAPI for 3 h at RT, washed in PBS, and mounted in Confocal-Matrix^®^ under a coverslip.

### Microscopy

High-resolution microscopy images were obtained on a Nikon Eclipse TE2000-U fluorescence microscope (Tokyo, Japan) equipped with a digital camera (Vosskühler COOL-1300), Lucia G or NIS Elements AR software, and a C1 confocal laser scanning unit with Helium/Neon (He/Ne; 543 nm) and Argon (488 nm) laser, governed by EZ-C1 or NIS-Elements AR software. The objectives (Nikon) used were a dry 4 × (NA, 0.13), dry 10 × (NA, 0.3), dry 20 × (NA, 0.5), dry 40 × (NA, 0.75), 40× oil-immersion (NA, 1.0), and 60× oil-immersion (NA, 1.4). For imaging of whole neurons, a Zeiss 510 META confocal laser scanning microscope was employed using an Argon laser (488) and 40× oil-immersion (NA, 1.3) objective.

### Algorithm-Based Spine Morphology Analysis

Secondary and tertiary segments (≥25 μm) of the apical (middle-third) dendritic subregions of pyramidal cells from hippocampal CA1 and CA3 (Bregma level from −1.58 to −2.03 mm) and two cortical areas, the parietal association cortex (ACTX; Bregma level from −1.46 to −2.06 mm) and the primary somatosensory cortex (SCTX; Bregma level from −1.34 to −2.06 mm) were imaged with a voxel size of 0.05 × 0.05 × 0.20 μm in x-y-z directions. Image stacks were processed using 3D blind deconvolution (Autodeblur Software) to improve signal-to-noise ratio and spatial resolution. Analysis of spine density, length, and shape was performed by algorithm-based, semi-automated evaluation of spine morphology using 3DMA-Neuron software as previously detailed (Sündermann et al., 2012). Spine morphology was classified into three groups: “stubby,” “mushroom” and “thin” based on the ratio of head to neck diameter and the ratio of spine length to neck diameter as described in Koh et al. (2002). The software developers based their criteria on a thorough EM study by Harris et al. (1992). Data are represented using nonlinear curve fitting with allometric 1 function: y = a*x^b^ (y = dependent variable; x = independent variable; a = coefficient; b = power).

For the 3D reconstruction of hippocampal neurons, pyramidal cells were imaged in 6–10 individual but overlapping stacks with a voxel size of 0.30 × 0.30 × 0.44 μm in x-y-z directions. Image stacks were converted into 8-bit grayscale .tif files using ImageJ software and then stitched to generate a single stack using VIAS software (Computational Neurobiology and Imaging Center, Mt. Sinai School of Medicine, New York, NY, USA). Three-dimensional reconstruction of whole neurons was performed using Neuromantic software (University of Reading, Reading, UK), which allows analysis of neuronal morphology after semi-automated tracing of dendritic processes, as previously described (Golovyashkina et al., 2014). Target neurons for all assessments were identified by anatomical location and cell morphology.

### Determination of Neuron Density

For the determination of neuron numbers in cortical slices, a machine learning-based approach for fast and unbiased analysis was used (Penazzi et al., 2014). Images from coronal sections between Bregma −0.94 mm and −2.06 mm (Paxinos and Franklin, 2004) were acquired by epifluorescence microscopy and processed through a series of macros programmed for Fiji (Schindelin et al., 2012) consisting of the following plug-ins: Hybrid Median Filter 2D (Christopher Philip Mauer, Vytas Bindokas), Anisotropic Diffusion (Vladimir Pilny, Jiri Janacek), and enhance local contrast (CLAHE; Stephan Saalfeld). For machine learning, several classifiers were trained and applied with the Advanced Weka Segmentation Plug-in (Ignacio Arganda-Carreras, Albert Cardona, and Verena Kaynig).

### Determination of Cortical Thickness, and Thickness of the Dentate Gyrus Granule Layer

The thickness of the neocortex was determined on the coronal sections of the same Bregma levels as those used for the determination of neuronal density. The thickness of the dentate gyrus (DG) was determined using coronal sections between Bregma −1.58 mm and −1.94 mm (Paxinos and Franklin, 2004) obtained from EGFP-expressing mice. The thickness was measured at the same position of the supra- and infra-pyramidal blade using the Fiji image processing package (Schindelin et al., 2012).

### Western Blot Analyses

Brain tissue was homogenized in 4 ml of RIPA buffer (50 mM Tris-HCl, 150 mM NaCl, 1 mM EDTA, 1% NP-40, 0.5% sodium deoxycholate, and 0.1% SDS, pH 8.0) per gram brain in the presence of protease and phosphatase inhibitors (1 mM PMSF, 10 mg/ml each of leupeptin and pepstatin, 1 mM EGTA, 1 mM sodium orthovanadate, 20 mM sodium fluoride, and 1 mM sodium pyrophosphate), sonicated (10–15 pulses), and centrifuged for 10 min at 13,000× *g* at 4°C. The supernatant was frozen and stored at −80°C. Protein concentration was determined using a bicinchoninic acid (BCA) protein assay kit (Thermo Fisher Scientific, Waltham, MA, USA). The samples were subjected to SDS–PAGE and transferred to Immobilon-P membranes (Millipore) followed by immunoblotting. Protein bands were detected using enhanced chemiluminescence with SuperSignal West Dura extended duration substrate (Thermo Fisher Scientific, Waltham, MA, USA) according to the manufacturer’s protocol. Quantification of the blots was performed with Gel-Pro Analyzer 4.0 (Media Cybernetics L.P., Baltimore, MD, USA) or by FusionCapt Advance (Vilber Lourmat, France).

### Statistical Analyses

For dendritic spine analysis, a generalized linear model was used, which is a flexible generalization of ordinary linear regression that allows for other than a normal distribution of the dependent variable and does not assume a linear relationship between the response variable and the model parameter. We have validated our generalized linear model (GZLM) with a finite sample corrected AIC (AICC). The normality of the data set was assessed by the Shapiro–Wilk test. All other measurements were statistically evaluated using the Student’s *t*-test or ANOVA, as depicted in the figure legends.

Statistical analyses were performed using Origin 7.0 (Microcal Software, Northampton, MA, USA) and SPSS Statistics 23 and 24 (Armonk, NY, USA: IBM Corp).

## Results

### APP_SDL_ Mice Accumulate Aβ40 and Aβ42 During Their Lifetime

**Figure 1:**
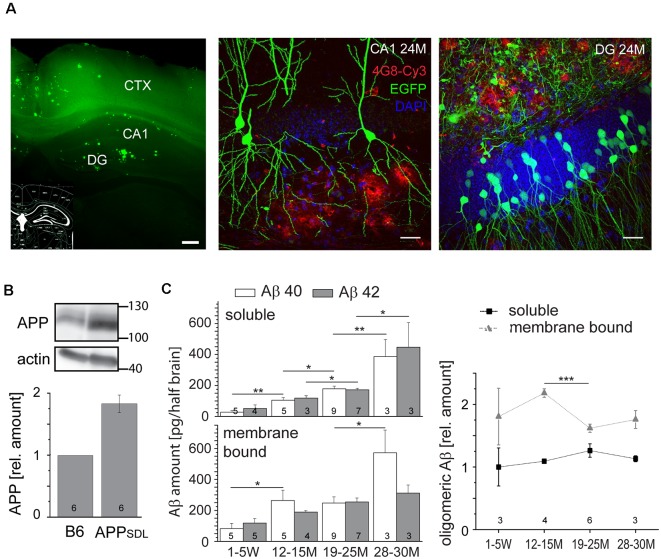
Amyloid beta (Aβ) precursor protein (APP_SDL_) mice accumulate Aβ40/Aβ42 during life with relatively stable amounts of the oligomeric forms. **(A)** Thioflavin S staining of a coronal vibratome section from a 24-month-old APP_SDL_ mouse, left. Confocal laser scanning micrographs showing Aβ deposits (4G8 staining, red) in the hippocampus from coronal sections of APP_SDL_ mice expressing EGFP in a population of hippocampal pyramidal neurons within the CA1 subfield, middle, and granule cells within the dentate gyrus (DG), right. Cell nuclei were visualized using DAPI. **(B)** The expression level of APP in B6 and APP_SDL_ animals. Immunoblot (top) of forebrain lysates from 24 months old mice detected with an anti-APP antibody that recognizes both human and mouse APP. Staining against actin is shown as loading control. Quantitation (bottom) of the APP signal was performed relative to actin and the value for B6 animals was set as 1.0. **(C)** Amount of soluble and membrane-bound human Aβ40 and Aβ42 (left), and the relative amount of oligomeric Aβ (right) from APP_SDL_ animals of different ages. Quantitation was performed by ELISA after the sequential extraction of brain lysates. The number of mice for each condition is indicated in bars (left) or right above the x-axis (right). Data represent mean ± SEM; the number of mice per genotype is shown at the graphs. Data were analyzed using Student’s *t*-tests to address potential alterations between adjacent time points within the same condition and considered to be significantly different at *p < 0.05, **p < 0.01 and ***p < 0.001. Scale bar, 200 μm **(A)** left, 20 μm **(A)** middle, right.

To analyze the chronic effects of Aβ during aging, we used transgenic mice that produce moderate levels of Aβ and develop plaques only at old age (Blanchard et al., 2003). APP_SDL_ mice express a human *APP* splice variant with a combination of three FAD-linked mutations (Swedish, Dutch, and London). Transgene expression is governed by the *PDGFB* promoter that confers neuron-specific expression (Sasahara et al., 1991) from as early as embryonic day 15 (E15; Hutchins and Jefferson, 1992). Embryonic cortical cultures from these mice produce equimolar amounts of Aβ40 and Aβ42 in the picomolar range (Leschik et al., 2007). Mice were maintained on a C57BL/6J background and compared with nontransgenic littermates (designated as B6). We first confirmed brain parenchymal plaque formation on coronal sections stained by thioflavin S and further validated by 4G8 antibody staining. The Aβ deposits appeared sparsely first at 18 months, as previously described (Blanchard et al., 2003). The hippocampal DG was the most affected region; however, with the increasing age of the mice, plaques also appeared in the CA1 and CA3 regions and spread partially within the cortex (Figure 1A).

Next, we analyzed the level to which APP_SDL_ mice expressed the human APP695 transgene. We observed a doubling of the total APP amount by semi-quantitative western blotting using a monoclonal antibody that detects both endogenous mouse and transgenic human APP (Figure 1B); there was an about equal expression of mouse and human APP. The expression of APP did not change throughout the life of the mice (data not shown). To determine the potential accumulation of Aβ species during aging, we quantified Aβ levels by ELISA. Because interaction with membranes may modulate amyloid aggregation and cytotoxicity (Evangelisti et al., 2016), we performed a sequential extraction of soluble and membrane-bound Aβ40 and Aβ42 before quantitation. We observed that the amount of aqueous soluble and membrane-bound (detergent-soluble) Aβ40 and Aβ42 gradually increased with age (Figure 1C, left). To test for the presence of oligomeric forms of Aβ and determine whether they accumulate in the same gradual manner, we employed an additional ELISA that is highly specific for the oligomeric form of the Aβ peptide. We observed that the levels of oligomeric Aβ were slightly higher in the membrane-bound fraction, consistent with the role of membrane components in promoting Aβ oligomerization (Wakabayashi and Matsuzaki, 2009; Figure 1C, right). The portion of oligomeric Aβ did not show an obvious age-dependent increase. Indeed, the amount of oligomeric membrane-bound fraction of Aβ even exhibited a significant drop at the time of plaque formation. Please note that Aβ peptides, the main components of senile plaques, predominantly form regular fibrils within the plaques, which are highly insoluble (Chen et al., 2017). Thus, they are not in the extracted fractions (both soluble and membrane-bound) that were used for the ELISAs.

These data indicate that APP_SDL_ mice accumulate predominantly monomeric Aβ during their lifetime, while the portion of the potentially most cytotoxic species (i.e., o-Aβ) remained constant.

### APP_SDL_ Mice Do Not Show Evidence of Neuron Loss or Increased Tau Phosphorylation

**Figure 2:**
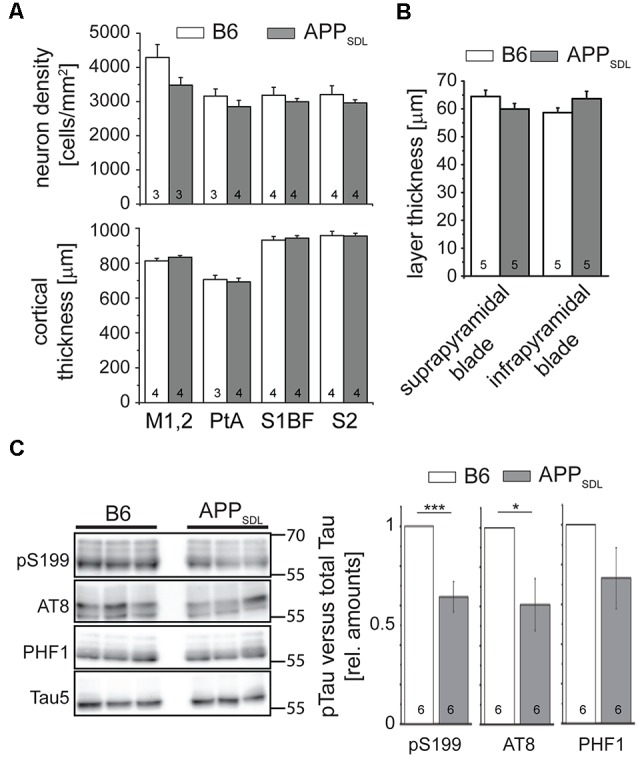
APP_SDL_ mice do not show signs of neuron loss or tau pathology. **(A)** Neuron cell body density (top) and cortical thickness (bottom) in different cortical regions of APP_SDL_ and B6 mice at 21 months of age. Quantitation of neuronal density was performed using a machine learning approach (see Supplementary Figure S1). Sections were sampled through the complete volume of the respective brain regions: M1, 2 = primary motor cortex (*n* = 11 B6, 11 APP_SDL_); PtA = parietal association cortex (*n* = 14 B6, 20 APP_SDL_); S1BF = primary somatosensory cortex, barrel field (*n* = 32 B6, 41 APP_SDL_); S2 = secondary somatosensory cortex (*n* = 20 B6, 43 APP_SDL_). **(B)** Thickness of the suprapyramidal and infrapyramidal blades of the DG. Five averaged measurements from 10 mice/genotype were performed on epifluorescent images of coronal sections of EGFP expressing mice. **(C)** Phosphorylation profile of tau at disease-relevant sites in APP_SDL_ and B6 mice at 20–25 months of age. Quantitation of signals was performed relative to total tau (Tau5 staining) and values for B6 animals were set as 1.0. Representative western blots are shown left. Data represent mean ± SEM; the number of mice per genotype is shown in the bars. Data were analyzed using Student’s *t*-test, and considered to be significantly different at *p < 0.05 and ***p < 0.001.

AD is characterized by a neurodegenerative triad of synaptic changes, dendritic simplification, and neuron loss (Wu et al., 2010; Bakota and Brandt, 2016), where the death of neurons is thought to occur last during disease and in a tau-dependent manner. To analyze whether the chronic presence of Aβ causes loss of neurons in the neocortex, we used a machine-learning approach for unbiased analysis and determined the density of neuronal cell bodies in aged APP_SDL_ and age-matched B6 mice (Figure 2A and Supplementary Figure S1). As neurons in different cortical areas may differ concerning their susceptibility to age-related changes, four spatially and functionally distinct regions were subjected to analysis. To avoid bias due to tissue shrinkage, we also measured the thickness of the tissue of the same cortical regions. We did not observe a significant difference between the two genotypes, either in cell density or in the thickness of the cortex, indicating that APP_SDL_ mice do not exhibit neuron loss (Figure 2A). We also tested for neuron loss in the DG since plaque formation in these mice affects this hippocampal subfield earliest. We determined the thickness of the granule cell layer of the supra- and infrapyramidal blade as an indicator of neuronal degeneration. This area also did not show reduced thickness in APP transgenic mice. In both genotypes, the thickness of the layer was approximately 60 μm, which agrees with published results (Figure 2B; Amaral et al., 2007).

To determine whether the mice develop early signs of tau pathology, we undertook a semi-quantitative western blot approach to identify a potential increase in tau phosphorylation at sites previously been shown to have increased levels of tau phosphorylation in AD patients (Buée et al., 2000). We observed that tau phosphorylation decreased at S199 and the AT8 epitope in old APP_SDL_ mice compared with age-matched, nontransgenic littermates, indicating that the chronic presence of Aβ alone does not induce increased phosphorylation of endogenous mouse tau at AD-relevant sites (Figure 2C). While it is generally believed that high concentrations of Aβ induce hyperphosphorylation of tau during disease (Hanger et al., 2007), we have previously shown in a cell culture system that nanomolar concentrations of secreted Aβ can induce a decrease in tau phosphorylation at selected sites (Golovyashkina et al., 2015). In support, it was also shown that Aβ can activate phosphatases *via* NMDA glutamate receptors (Shankar et al., 2007), which can act on certain tau phosphoepitopes (Wei et al., 2002; Rahman et al., 2006).

### Hippocampal Pyramidal Neurons Respond to Elevated Aβ by Decreasing Spine Density, Whereas Cortical Neurons Are More Resilient

Dendritic spines are considered to be the major loci of excitatory synaptic plasticity and function. They can respond to developmental challenges, novel experiences, and noxious stimuli. Higher than physiological Aβ levels pose great demands on dendritic spines (Terry et al., 1991; Shankar et al., 2008). Therefore, it is important to determine how chronic Aβ levels affect pyramidal cells within different brain regions during a lifetime and if the gradually increased amounts of Aβ exert a progressive burden on dendritic spines. To evaluate spine changes in age- and brain region-dependent manner, we employed algorithm-based image analyses of fluorescent neurons from 3D image stacks. To visualize neurons of interest, we crossed APP_SDL_ mice with those of the GFP M line (Feng et al., 2000) in which EGFP labels a small population of neurons in various brain regions, including the hippocampus and cortex. In these mice, EGFP expression is governed by the *Thy1* promoter, which drives expression during early postnatal life (Morris, 1985) and sufficiently labels some neurons already in 5-week-old mice. EGFP expression did not affect plaque formation as double-transgenic EGFP/APP_SDL_ mice showed a similar temporal and regional distribution of Aβ plaques as that previously described (Figure 1A, middle, right).

For the analysis, we chose four age groups to cover a broad spectrum of developmental stages representing adolescence (5 weeks of age), young adult (3 months of age), middle-age (15 months of age; shortly before plaque formation), and old (24 months of age; presenting with Aβ plaques). We sampled from two hippocampal (CA1 and CA3) and two cortical (parietal association cortex: ACTX, and primary sensory cortex; SCTX) brain areas to obtain information about developmental and coping strategies of different brain regions and subregions. Finally, we extended our analysis to both sexes to determine whether either sex has a potentially higher susceptibility to Aβ-mediated synaptic changes. It has been reported that there is pronounced instability in pre- and postsynaptic structures within the vicinity of amyloid plaques (Liebscher et al., 2014), which can markedly influence spine parameters. However, in our mouse model, only moderate amounts of plaques are present, and only in the old mice. Consequently, our analysis excluded possible changes in the spine parameters in the vicinity of plaques.

**Figure 3:**
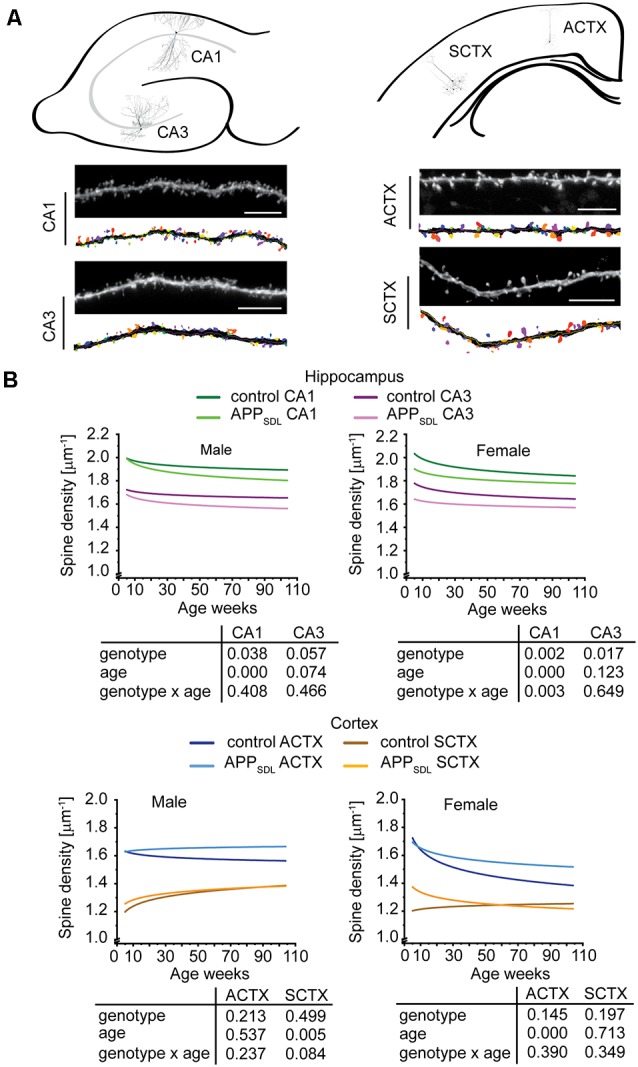
Spine density trajectories of hippocampal and cortical pyramidal neurons. **(A)** Schematic representation of the hippocampus (top, left) and cortex (top, right) with the respective exemplary pyramidal neurons that were analyzed. Representative high-resolution images of dendritic segments from the respective neurons after blind deconvolution and 3DMA analysis (bottom) are shown. **(B)** Spine density trajectories of hippocampal CA1 and CA3 neurons and cortical parietal association (ACTX) and primary sensory (SCTX) neurons from EGFP and APP_SDL_ transgenic mice of different age. Please note that the age of mice had been displayed in weeks to represent it as scaled time intervals. Statistical data were assessed using a generalized linear model from six to seven mice per condition (see the total number of dendritic segments used for each condition in Supplementary Table S1). The *p*-value of the genotype, age effects, and their interaction are shown below the graphs. Scale bar, 10 μm. The distribution of individual data points are presented in Supplementary Figure S2.

We first quantified dendritic spine densities under different conditions because they could serve as indicators of the extent of alterations in synaptic connectivity in different brain regions (Segal, 2005; Harms and Dunaevsky, 2007; Figure 3A). To determine changes occurring over time, we analyzed our data using a generalized linear model (GZLM). As an extension to a traditional general linear model like a multivariate ANOVA, it allows for other than a normal distribution of the dependent variable (Choi et al., 2018) and does not assume a linear relationship between the response variable and the model parameter, i.e., in this case the spine parameters and the age of mice (Faraway, 2010). The results were plotted as fitted spine density trajectories (Schumann et al., 2010; Figure 3B, Supplementary Figure S2). In the hippocampus, GZLM analysis revealed a significant genotype effect at most conditions (Figure 3B, top), where spine density trajectories representing data form CA1 and CA3 pyramidal neurons of APP_SDL_ mice ran below the trajectories of the respective data from control mice. This confirmed the negative effect, i.e., the spine loss initiated by Aβ on hippocampal principal neurons. In the CA1 subfield, the genotype effect was observed in both male and female mice, indicating that the hippocampus in both sexes is sensitive to elevated Aβ levels. Spine density trajectories also revealed a decrease during the lifetime of the mice, resulting in a significant age effect. The interaction between genotype and age could only be observed for the CA1 pyramidal neurons of female mice, implying that age and spine reduction by Aβ are generally not interdependent in the hippocampus.

Interestingly, the results were different for cortical regions. In most cases, the spine density trajectory depicting data from APP_SDL_ mice ran higher than the trajectory for the respective data from control mice; however, the difference did not result in a significant genotype effect in the two regions analyzed (Figure 3B, bottom).

Taken together, the data indicate that, although increased amounts of Aβ present a challenge for the dendritic spines throughout aging, under our conditions no extensive alterations occurred at the spine level. Moreover, hippocampal principal neurons responded to elevated Aβ levels in the brain with a loss of spines, whereas cortical neurons were less responsive, perhaps even showing an inverse reaction. Our data provide evidence that these changes are present in both sexes. Furthermore, they do not progress with age. The different effects of Aβ on the spine density trajectories in the hippocampus and the cortex may suggest a potential compensatory adaptation of pyramidal cells in the cortex.

### Elevated Amounts of Aβ Induce Region-Specific Changes in Spine Morphology, Including a Shift From Mushroom Spines to Stubby Spines in the CA1 Subfield

Individual spines also develop morphological adaptations during maturation and exhibit changes during learning or noxious insult (Berry and Nedivi, 2017). Thus, the question arises as to whether elevated Aβ levels influence spine morphology in the brain areas analyzed.

To approach this question, we classified spines into three morphological categories, namely, “mushroom,” “stubby,” and “thin” (Harris et al., 1992), which are thought to represent functionally distinct entities. The analysis was performed by an algorithm-based method using cLSM image stacks as previously described (Sündermann et al., 2012).

**Figure 4:**
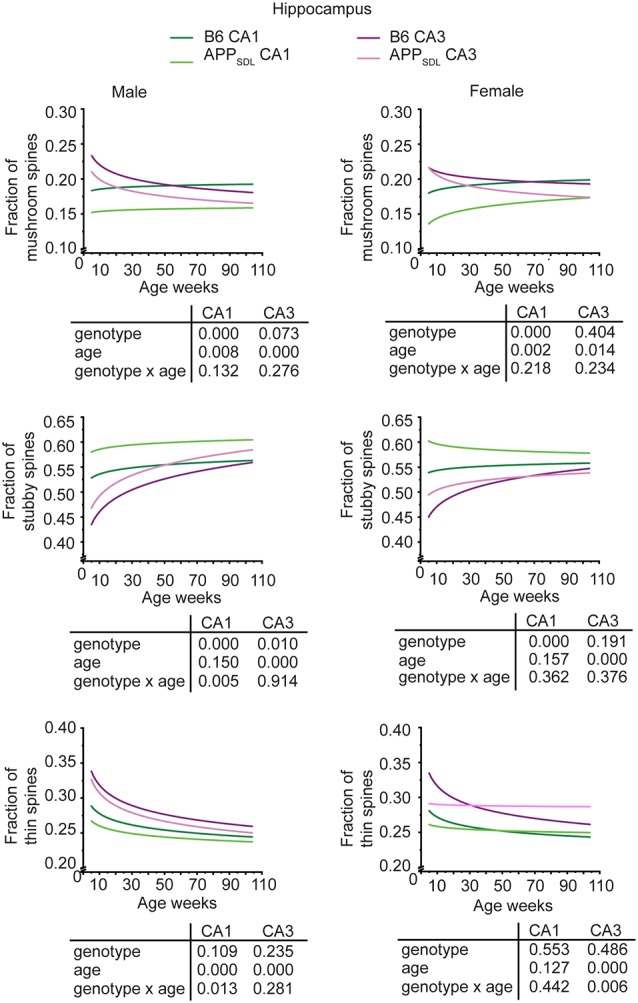
Spine morphing in APP_SDL_ mice is differentially affected in hippocampal subfields of the two sexes. Trajectories representing the fractions of different spine classes from CA1 and CA3 pyramidal neurons in APP_SDL_ transgenic and control B6 mice of different ages as determined by algorithm-based classification. Please note that the age of mice had been displayed in weeks to represent it as scaled time intervals. Statistical data were assessed using a generalized linear model from six to seven mice per condition (see the total number of dendritic segments used for each condition in Supplementary Table S1). The *p*-value of the genotype, age effects, and their interaction are shown below the graphs. Distribution of individual data points are presented in Supplementary Figures S3, S4.

**Figure 5:**
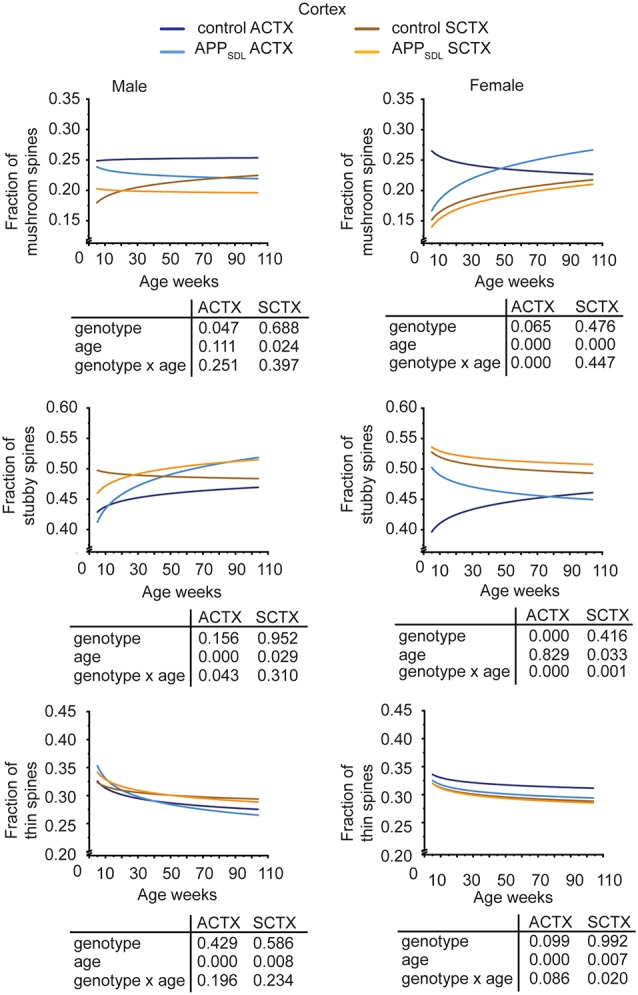
Spine morphing in APP_SDL_ mice is differentially affected in cortical brain regions of the two sexes. Trajectories representing the fractions of different spine classes from parietal association (ACTX) and primary sensory (SCTX) pyramidal neurons in APP_SDL_ transgenic mice and control B6 mice of different ages as determined by algorithm-based classification. Please note that the age of mice had been displayed in weeks to represent it as scaled time intervals. Statistical data were assessed using a generalized linear model from six to seven mice per condition (see the total number of dendritic segments used for each condition in Supplementary Table S1). The *p*-value of the genotype, age effects, and their interaction are shown below the graphs. Distribution of individual data points are presented in Supplementary Figures S5, S6.

Region-specific differences developed with respect to the mushroom and stubby spine types. In the hippocampus, we observed a genotype effect with both mushroom and stubby spines in the CA1 subfield of both sexes (Figure 4; Supplementary Figure S3). In this subfield, the respective trajectories depicting spine fractions exhibited a shift between the ratios of the two spine classes. Specifically, mice with elevated amounts of Aβ showed an increase in the stubby fraction of spines compared with the mushroom fraction. A significant increase in the fraction of stubby spines was also observed for male mice in the CA3 subfield, while female mice did not exhibit a significant difference (Figure 4; Supplementary Figure S4). A decreased fraction of mushroom spine was observed in the ACTX of male mice, as evidenced by the genotype effect after a GZLM analysis. In contrast, the SCTX was completely unresponsive to the presence of increased levels of Aβ in both sexes (Figure 5; Supplementary Figures S5, S6).

A surprising observation was the change in the association cortex of female mice, where the proportion of mushroom spines started low in adolescent APP_SDL_ mice, as also observed in the other brain areas. However, during aging, this spine type fraction showed an upward trajectory, overtaking the proportion of the mushroom spine fraction in control animals at later stages (Figure 5, Supplementary Figure S5).

In most brain regions analyzed, and for both genotypes and sexes, the trajectories of the fraction of thin spines showed a decrease with age. This decline during the lifetime of the mice is significant as the GZLM analysis showed a significant age effect (Figures 4, 5). These results confirmed that there is an age-related loss of synaptic plasticity in older mice as previously described (Xu et al., 2018). However, we did not observe a significant genotype effect for the thin spines, further corroborating that the decrease in thin spines throughout the lifetime of mice is due to age-related, rather than Aβ-related, alterations in cell function (Figures 4, 5).

Taken together, the data indicate that Aβ induces a clear shift in spine morphing from mushroom to stubby in the hippocampal CA1 region, whereas the SCTX was completely unresponsive. These data suggest that elevated levels of Aβ direct spine morphology towards lower compartmentalization in the affected brain areas. Furthermore, some of these morphological changes differ between sexes in a region-specific manner.

### Transgenic APP Expression Does Not Differ Between the Hippocampus and Cortex

**Figure 6:**
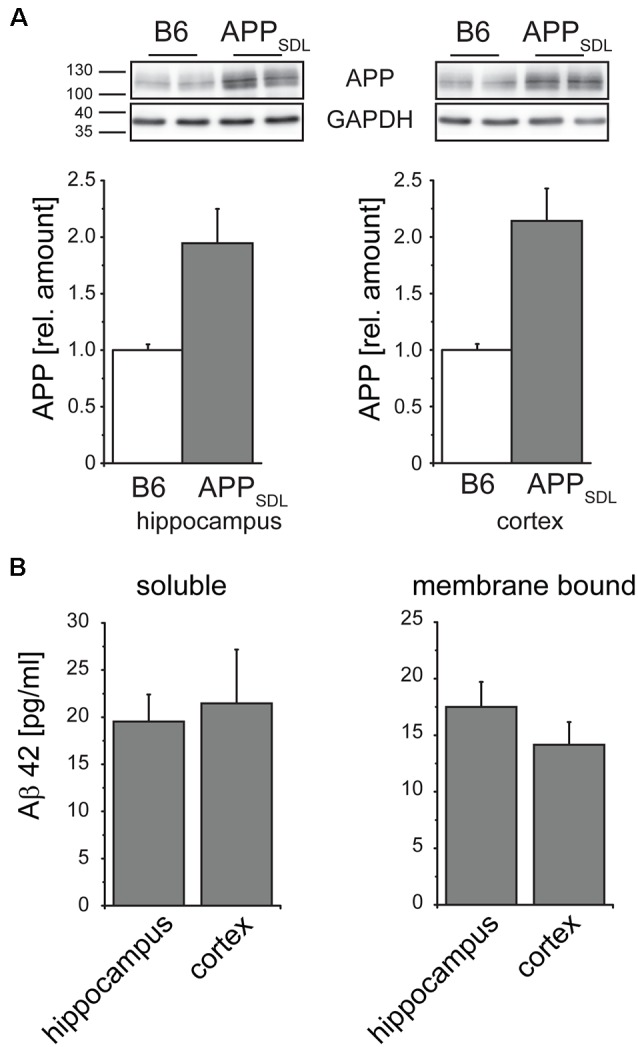
Relative amounts of APP in the hippocampus and cortex do not differ in APP_SDL_ mice. **(A)** Representative immunoblots from cortical and hippocampal brain lysates (top). Graphs showing the relative amounts of APP expression in B6 and APP_SDL_ animals at 20–28 months of age between the two genotypes. Quantitation of signals was performed from 9 to 10 mice per genotype (bottom). Data were calculated relative to GAPDH and values for B6 animals were set as 1.0. **(B)** Quantification of the Aβ42 concentration within the soluble and membrane-bound fraction of hippocampal and cortical samples. Data originate from eight APP_SDL_ mice at 23–28 months of age. Data represent mean ± SEM.

To address if the diverse effects on dendritic spines are due to different expression levels of the transgene between the cortex and hippocampus of APP_SDL_ mice, we performed APP-targeted western blot analysis separately on both brain regions. We again observed a one-fold higher expression of the transgene, which was similar in both brain regions (Figure 6A). As APP processing or Aβ clearance can differ within the brain, we also determined the level of Aβ42, which is considered to be the main synaptotoxic species (Walsh and Selkoe, 2007). We did not see a significant difference either in the soluble or in the membrane-bound form of Aβ42 (Figure 6B), indicating that the differential spine alterations in the analyzed brain regions were not due to differences in the levels of APP or Aβ42.

### Basal Dendrites of CA1 Pyramidal Neurons Exhibit Dendritic Simplification in Old APP_SDL_ Mice

Dendritic simplification, an aspect of the neurodegenerative triad during AD, has been investigated substantially less than spine changes and neuronal death. However, changes in dendritic arborization have the potential to markedly influence synaptic connectivity.

**Figure 7:**
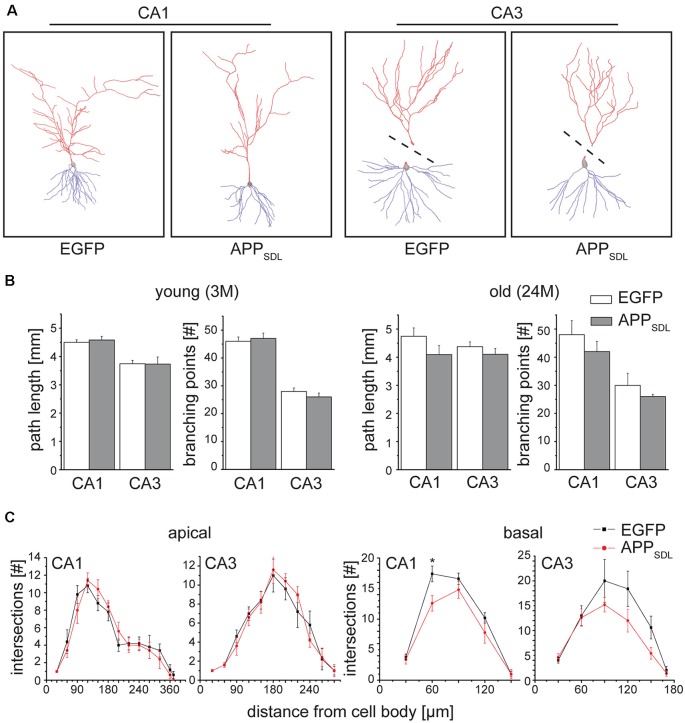
Old APP_SDL_ mice exhibit dendritic simplification on basal dendrites in the hippocampus. **(A)** Representative reconstructed neurons from CA1 and CA3 regions of the hippocampus from 24 months old control and APP_SDL_ transgenic mice. Apical arbor is indicated in red, basal arbor in blue. Dashed lines indicate that apical and basal arbor were imaged and reconstructed separately due to the cutting angle during slicing. **(B)** Path length and the number of branching points of hippocampal pyramidal cells from young (3 months) and old (24 months) control and APP_SDL_ transgenic mice. **(C)** Sholl analysis of apical and basal arbors of hippocampal pyramidal cells from old (24 month) control and APP_SDL_ transgenic mice. CA1 pyramidal neurons from old APP_SDL_ transgenic mice show dendritic simplification on basal dendrites in comparison to control animals *F*_(1,4)_ = 24.14; *p* = 0.0080. Data represent mean ± SEM. Neurons were analyzed from five to six mice per genotype. Data were analyzed using repeated-measures ANOVA with Sidak *post hoc* test and considered to be significantly different at *p < 0.05.

We determined changes in the gross morphology of neurons from young (3 months) and old (24 months) EGFP/APP_SDL_ mice and compared them to age-matched EGFP-expressing controls. We focused our investigation on the hippocampus, which we found to be the most susceptible to increases in Aβ levels, as indicated by changes in spine parameters. CA1 and CA3 pyramidal neurons were imaged in high-resolution tile z-stacks and reconstructed in 3D (Figure 7A). The morphological parameters total path length and the number of branching points were determined. We did not observe a statistically significant difference between neurons from APP_SDL_ mice and those from control animals; however, there was a general trend towards dendritic simplification in old APP_SDL_-expressing mice (Figure 7B). To gather detailed information about potential region-specific morphological changes induced by Aβ, we employed Sholl analysis to measure dendritic field density and structure (Sholl, 1953). Compared with age-matched controls, we observed a reduced number of dendritic intersections in basal trees of the neurons, which reached significance at a distance of 60 μm from the cell body in CA1 neurons from old APP_SDL_ mice (Figure 7C). These data indicated that chronic Aβ levels induce dendritic simplification at old age in a regionally restricted manner and that dendritic simplification occurs before the loss of neurons.

### PSD-95 and Arc Respond to Chronic Aβ Production in Old APP_SDL_ Mice

**Figure 8:**
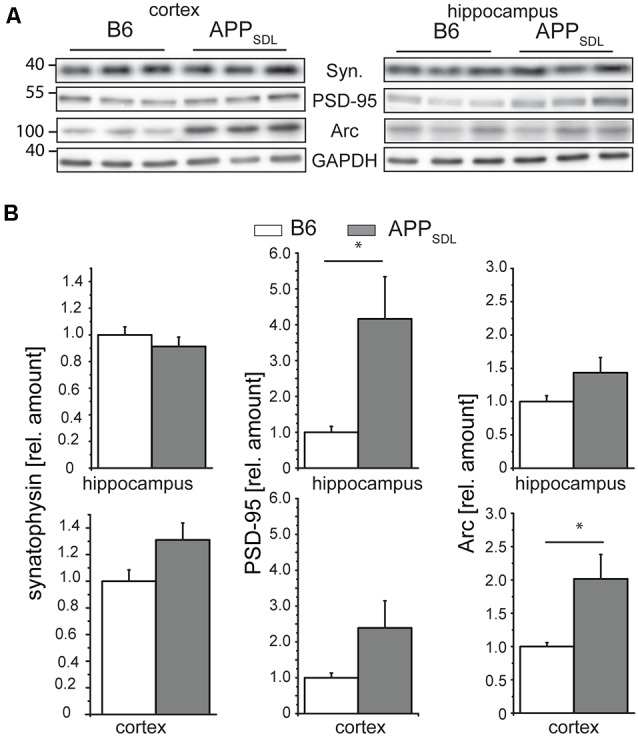
Synaptophysin, PSD-95 and Arc expression in response to chronic Aβ in old APP_SDL_ mice. **(A)** Representative immunoblots from cortical and hippocampal brain lysates. **(B)** Expression of synaptophysin (left panel), PSD-95 (middle) and Arc (right panel) in cortex and hippocampus of B6 and APP_SDL_ mice at 20–28 months of age. Quantitation of signals was performed from 9 to 10 mice per genotype. Data were calculated relative to GAPDH and values for B6 animals were set as 1.0. Data represent mean ± SEM and were analyzed using Student’s *t*-test. Data were considered to be significantly different at *p < 0.05.

Synaptophysin (major synaptic vesicle protein p38) is an established general marker for the quantification of synapses and synaptic integrity (Li et al., 2010). To determine whether the differential effect of Aβ on spine density in hippocampal and cortical neurons is also reflected in changes in synaptophysin levels, we measured the relative levels of synaptophysin in both brain regions by semi-quantitative western blotting (Figure 8A). We did not observe any significant changes in the level of synaptophysin between the genotypes in either brain region (Figure 8B, left). To test for potential changes in the postsynapse, we measured the relative amounts of PSD-95, a pivotal postsynaptic scaffolding protein in excitatory neurons (Kaizuka and Takumi, 2018). Surprisingly, we found that the levels of PSD-95 in APP_SDL_ mice were increased when compared with those of B6 controls, and this increase reached significance in the hippocampus (Figure 8B, middle top). As a more functional molecular readout, we also determined the level of the activity-regulated cytoskeletal protein Arc, which is crucial for every form of neuronal plasticity and can affect synaptic strength (Guzowski et al., 2000; Messaoudi et al., 2007; Peebles et al., 2010). The Arc protein levels showed a pronounced increase in the cortex of APP_SDL_ transgenic mice compared with those of control mice (Figure 8B, right), but the level of increase was lower in the hippocampus. As Arc has a key role in the consolidation of explicit and implicit forms of memory (Bramham et al., 2010), our data suggest that Arc levels may be region-specifically increased to strengthen memory consolidation and maintenance in the cortex.

Combined, these data indicate that the differential effect of Aβ on spine density in the hippocampus and cortex is not reflected on a molecular level by a change in the level of synaptophysin, a marker for presynaptic structure. Changes in the concentrations of PSD-95 and Arc may point to the existence of a compensatory effect in respective brain regions under our experimental conditions.

## Discussion

Aβ deposits are thought to play a causative role in AD (Hardy and Selkoe, 2002). Disagreement with the classical amyloid hypothesis was driven by several studies reporting that a proportion of individuals at risk of AD show intact cognition regardless of extensive accumulation of the Aβ peptide in their brain (Bennett et al., 2006; Arendt, 2009; Villemagne et al., 2013). Furthermore, analysis of the distribution of the histopathological lesions in AD patients has shown that tau inclusions show a higher correlation with cognitive impairment than amyloid plaques (Nelson et al., 2012). This raises the question as to the nature of the difference that makes a population resilient towards cognitive decline, despite the accumulation of Aβ, and how different brain regions adjust to the chronic presence of increasing amounts of Aβ during the long prodromal phase of the disease. As an example, Boros et al. (2017) analyzed the brains of control subjects with AD pathology and AD brains with dementia, and concluded that dendritic spines may provide cognitive resilience against AD. In light of this, we decided to analyze the effect of moderate concentrations of lifelong, chronic Aβ exposure, potential changes in oligomeric Aβ levels, and region-specific differences in spine parameters in a mouse model of amyloidosis.

Neurons and spines undergo structural adaptations throughout physiological aging that likely contribute to changes in their electrophysiological properties and cognition (Dickstein et al., 2013). However, AD already presents characteristics of a synaptopathology early during the disease process (Selkoe, 2002; Opazo et al., 2018), and synapse deficiencies may accelerate cognitive decline. In contrast, the magnitude of synaptic deficiencies appears not to be correlated with increasing Aβ levels, as otherwise the disease would be expected to turn symptomatic much earlier. Therefore, we aimed to analyze the extent to which established molecular and structural substrates of memory consolidation, i.e., the dendritic spines, develop alterations during the long prodromal stage of AD. For this, we used transgenic mice overexpressing low levels of the *APP* gene as a diagnostic tool for studying synaptic health. We reasoned that these mice might show a less pronounced, but more realistic view, of the alterations and adaptive potential of synaptic plasticity in different brain regions long before the development of symptomatic AD. We selected this model owing to the increasing concern about potential artifacts connected to high overexpression of the APP protein in many mouse models (Saito et al., 2016). We hypothesized that the directionality or magnitude of the alterations may differ in various brain regions, depending on the adaptive/compensatory capacity of the respective region.

Our findings were as follows: (1) Aβ species show a gradual accumulation throughout the life of transgenic APP_SDL_ mice, which is not paralleled by an increase in the levels of the oligomeric form. (2) Hippocampal pyramidal neurons respond to increased Aβ concentrations by lowering spine density and undergoing a shift in spine morphology from mushroom towards stubby spines; both changes occur mainly in the CA1 subfield regardless of the sex. (3) In contrast, the spine density trajectories of cortical pyramidal neurons differ in that they do not show a difference in spine density in response to increased Aβ exposure. (4) Reduced spine compartmentalization also occurred in cortical neurons analyzed, specifically in the ACTX of male mice. (5) Increased concentrations of PSD-95 and Arc in the hippocampus and cortex, respectively, point towards a compensatory mechanism initiated during the prodromal stage of amyloidosis.

Soluble oligomeric Aβ has been shown to induce the loss of dendritic spines in culture (Lacor et al., 2007; Shankar et al., 2008; Tackenberg and Brandt, 2009) and a reduction in spine density in APP transgenic mice (Koffie et al., 2009; Jung and Herms, 2012). Spine loss could be reversed either by restoring the cAMP/PKA/CREB signaling pathway (Smith et al., 2009) or by antibody-mediated neutralization of soluble Aβ (Shankar et al., 2007; Zago et al., 2012), suggesting that local modulation of spine number could be a physiological and reversible action mediated by Aβ and may not necessarily be neurotoxic in itself. Indeed, picomolar levels of Aβ were shown to enhance synaptic plasticity (Puzzo et al., 2008), supporting the physiological role of Aβ. Our data show that gradually increasing Aβ levels do not lead to steadily intensified defects in postsynaptic structures. Under certain conditions, we even observed that the greatest differences occurred between the genotypes at a very young age, e.g., the spine density in the female hippocampus (see Figure 3B).

Potential changes in plasticity may result in some brain regions becoming more resilient to the Aβ present, or even initiate various mechanisms to compensate for impaired signaling in other brain regions. Accordingly, Elman et al. (2014), using PET imaging, showed that one possibility for a compensatory mechanism, potentially reflecting brain plasticity in response to Aβ deposition, is an increased activity associated with more detailed memories that occur in some brain regions. However, Aβ-dependent neuronal hyperactivity is believed to contribute to circuit dysfunction at the early stages of AD (Zott et al., 2019). We have previously shown that APP_SDL_ mice present hippocampal hyperactivity at 17–18 months of age (Penazzi et al., 2017). Our current data indicate that neurons can effectively adapt through a long period of advancing age, even to a several-fold increase in Aβ concentrations, if the oligomeric proportion does not substantially change.

The unequivocal observation of this study is that the hippocampal CA1 subfield is the most affected brain region in APP_SDL_ mice of both sexes. Nevertheless, male APP_SDL_ mice aged 17–18 months do not have spatial memory deficits when plaques occur (Penazzi et al., 2017), probably due to sufficient brain plasticity. This supports the observation that behavioral symptoms appear at the late stages of neurodegenerative diseases, although morphological changes can be detected much earlier. Although an expected decrease in spine density was observed in the hippocampus, the cortical regions showed no or even a tendency for an opposite effect. As the latter observation does not reflect the general expectations, such data are rarely published. However, increased spine density has also been recognized through *in vivo* imaging of the layer 5 cortical neurons in the most often examined mice with amyloid pathology, the Tg2567 mice, at 12 months of age, a time when these mice are already developing plaques (Jung and Herms, 2012). This may be due to a different regulation of the expression of synapse regulating proteins in the hippocampus compared to the cortex. Mice encoding mutant APP and mutant PSEN1 (APdE9) have been shown to increase BDNF protein levels significantly during aging in cortical regions, but not in the hippocampus, compared to wild-type mice (Rantamäki et al., 2013).

We also showed that there were changes in synaptic connectivity by quantitative western blots for PSD-95 and the activity-regulated protein Arc at significant levels in a region-specific manner, whereas the level of the presynaptic protein, synaptophysin, showed no significant change. Accumulating evidence supports the functional importance of the early-expression *ARC* gene in regulating memory consolidation. Interestingly, AD patients express anomalous levels of the Arc protein (Rudinskiy et al., 2012). However, it is not clear what the effects are on the neurophysiology of AD-associated amyloidopathy. Arc interacts specifically with several effector proteins in different neuronal compartments such as dendritic spines and nuclear domains, and may bidirectionally regulate synaptic strength by distinct molecular mechanisms. This suggests that Arc may act as a master organizer of long-term synaptic plasticity, critical for information storage and cognition (Nikolaienko et al., 2018). In mice with high Aβ accumulation such as Tg2576 mice (Almeida et al., 2005), as well as in the postmortem brain (Proctor et al., 2010), the levels of PSD-95 were reported to be decreased and the degree of reduction correlated with the severity of dementia. However, Aβ pathology develops slowly in our mouse model and the presymptomatic phase is long. Therefore, increased PSD-95 levels, especially in the hippocampus where the spine alterations are more enhanced, could be indicative of potential compensatory mechanisms before the onset of severe symptoms.

Hippocampal and cortical neurons may differ in their sensitivity and adaptive response to increasing Aβ levels; moreover, cortical neurons might compensate for the loss of synaptic contacts in the hippocampus (or other brain regions that were not analyzed), thereby increasing memory consolidation and maintenance. Compared with those of AD patients with dementia, the levels of synaptic proteins such as synaptophysin and synaptopodin were also shown to be preserved in the brains of female subjects who presented with AD pathology but were resilient to cognitive decline (Arnold et al., 2013).

We showed that, in contrast to the distinct alterations in spine density within different brain regions, higher Aβ levels generally promote a lowering of the level of spine compartmentalization by inducing a decreased proportion of mushroom or increased proportion of stubby spine phenotype in the hippocampus and at specific conditions within the cortex. Using mathematical modeling, a recent study showed that shortening and widening of the necks should alter the electrical compartmentalization of the spines, leading to reduced postsynaptic potentials in spine heads, but not the soma, in APP×PS1-KI mice (Androuin et al., 2018). According to these observations, local EPSPs are likely to be lower at conditions where the stubby spine fraction in APP_SDL_ mice is increased compared to controls, which can affect the degree of potentiation of the postsynaptic cell. It would be interesting to address this experimentally in future studies. Taken together, our study highlights parallels and differences in spine plasticity mechanisms in cortical and hippocampal regions.

Females have a disproportionate occurrence of AD compared with males; however, the reason for this is not clear (Vina and Lloret, 2010). It is conceivable that changes in dendritic spines render females more susceptible to cognitive decline. Previous studies, performed mainly in rats, showed that gonadal hormones modulate dendritic spine densities (Chen et al., 2009). However, there is no consensus in the literature regarding the brain region affected or the stage of the estrus cycle that affects spine density (Shors et al., 2001; Peterson et al., 2015). To the best of our knowledge, no study to date has analyzed dendritic spine plasticity in both sexes throughout the life of mice. Our data do not support that certain brain regions or spine parameters in female mice are more markedly affected by increased Aβ levels than in male mice. Interestingly, in most cases where a genotype effect is observed in female mice, the largest differences in the trajectories of the two genotypes are found during adolescence and not in the aged brain. This suggests that the higher susceptibility of females to AD development is likely to be connected to other aspects of the disease rather than the sensitivity of dendritic spines to Aβ exposure.

The pathophysiology of AD is thought to develop over many years before the emergence of cognitive impairment and diagnosis of the disease. Recent disappointing clinical trial results raise the possibility that therapies may have a limited effect after neuronal degeneration has begun. This suggests that AD would be optimally treated in the presymptomatic stages of the disease. The possibility of analyzing changes in synaptic connectivity in a sensitive and temporospatially defined manner could provide a useful tool to guide the identification of potentially protective conditions or conditions that delay the onset of the disease. Our data provide evidence that, despite gradually increasing amounts of Aβ, some brain regions such as the primary somatosensory cortex, are more resilient to dendritic spine alterations than the hippocampus. The increased understanding of the mechanisms that drive this resilience could be of therapeutic relevance.

## Data Availability Statement

All datasets generated for this study are included in the article/Supplementary Material.

## Ethics Statement

The animal study was reviewed and approved by Niedersächsische Landesamt für Verbraucherschutz und Lebensmittelsicherheit 26029 Oldenburg Germany.

## Author Contributions

RB and LB designed the research. MVH, MR, NG, LP, WCP, and BD performed the research. MVH, MR, NG, LP, WCP, BD, FS, and LB analyzed the data. RB and LB wrote the article. All authors revised and approved the manuscript.

## Conflict of Interest

The authors declare that the research was conducted in the absence of any commercial or financial relationships that could be construed as a potential conflict of interest.
