# Supplementary figures and images for: Chronic Presence of Oligomeric Aβ Differentially Modulates Spine Parameters in the Hippocampus and Cortex of Mice With Low APP Transgene Expression
Source: Front Synaptic Neurosci. 2020 Apr 24;12:16. doi: 10.3389/fnsyn.2020.00016 (PMC7194154; doi:10.3389/fnsyn.2020.00016)

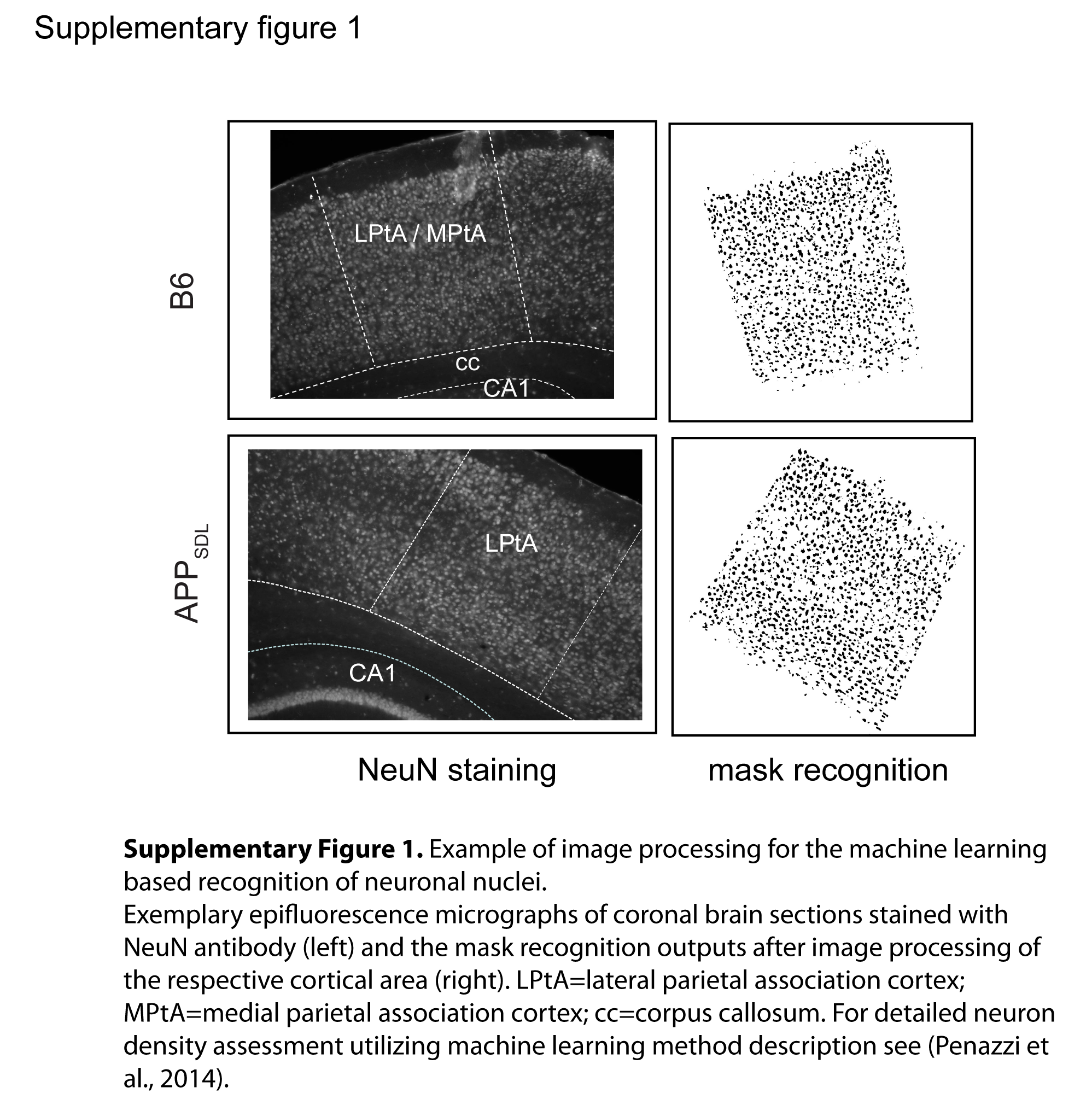

Supplement: Supplementary file 2 [file Image_1.TIF]

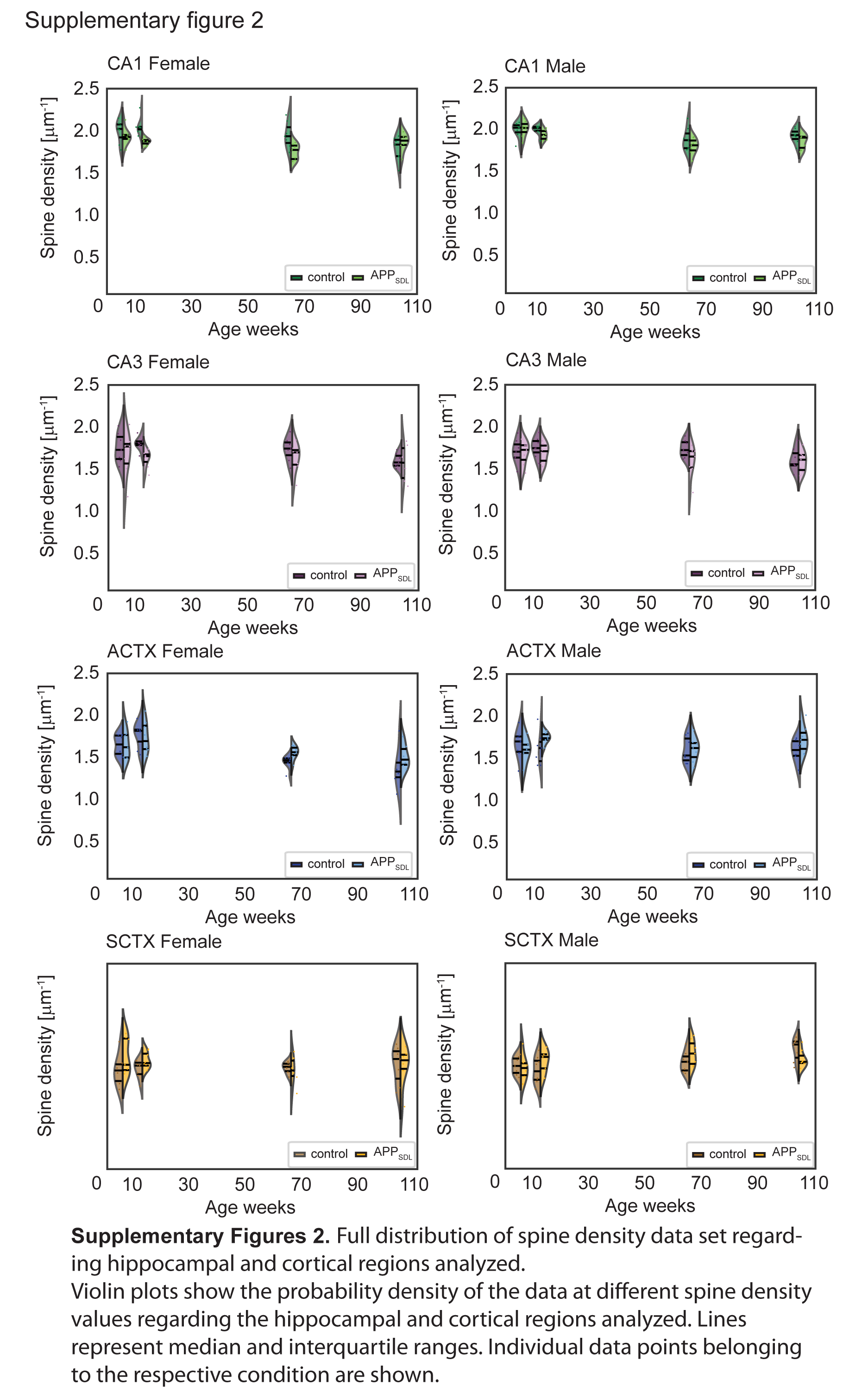

Supplement: Supplementary file 3 [file Image_2.TIF]

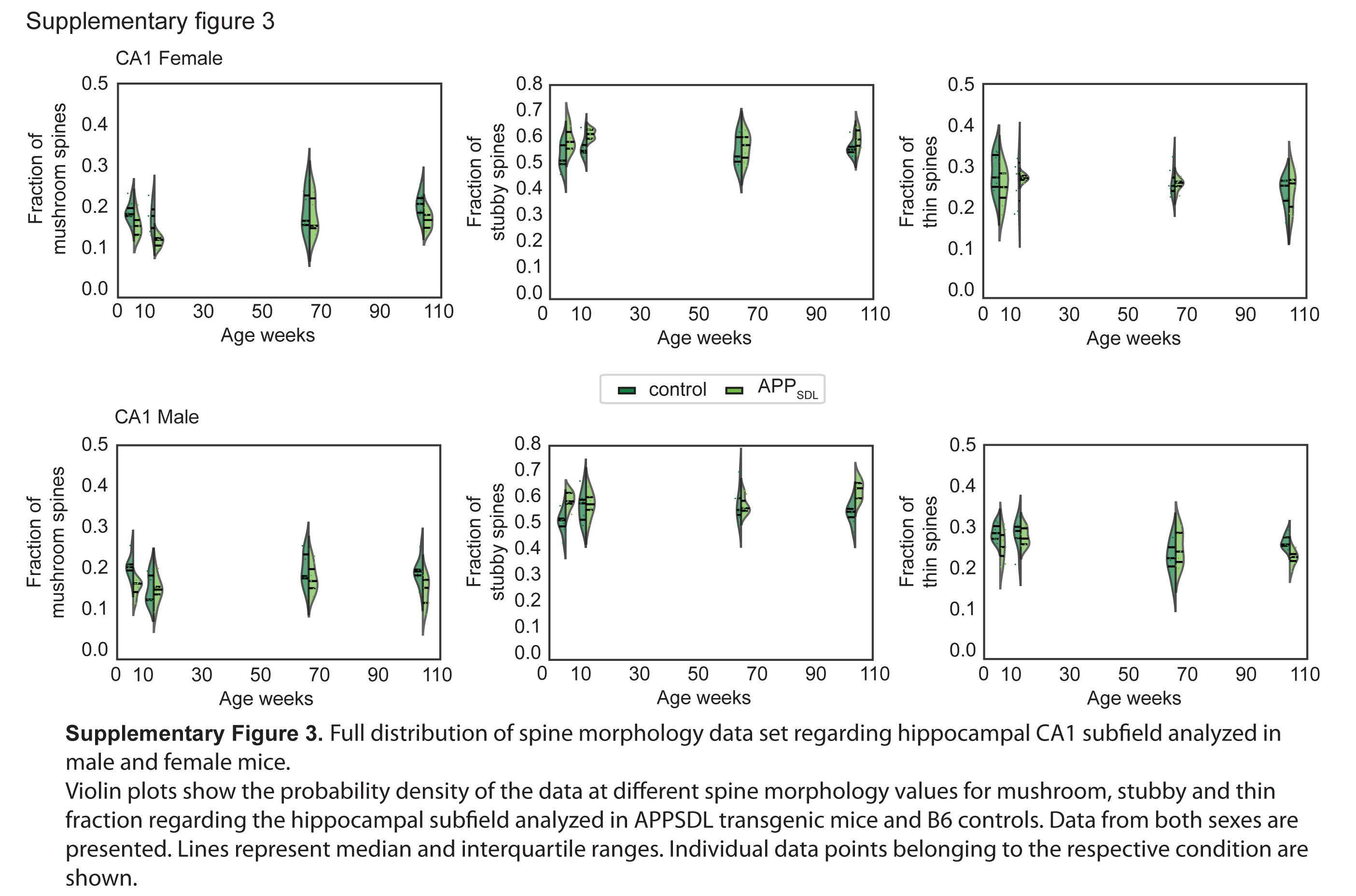

Supplement: Supplementary file 4 [file Image_3.TIF]

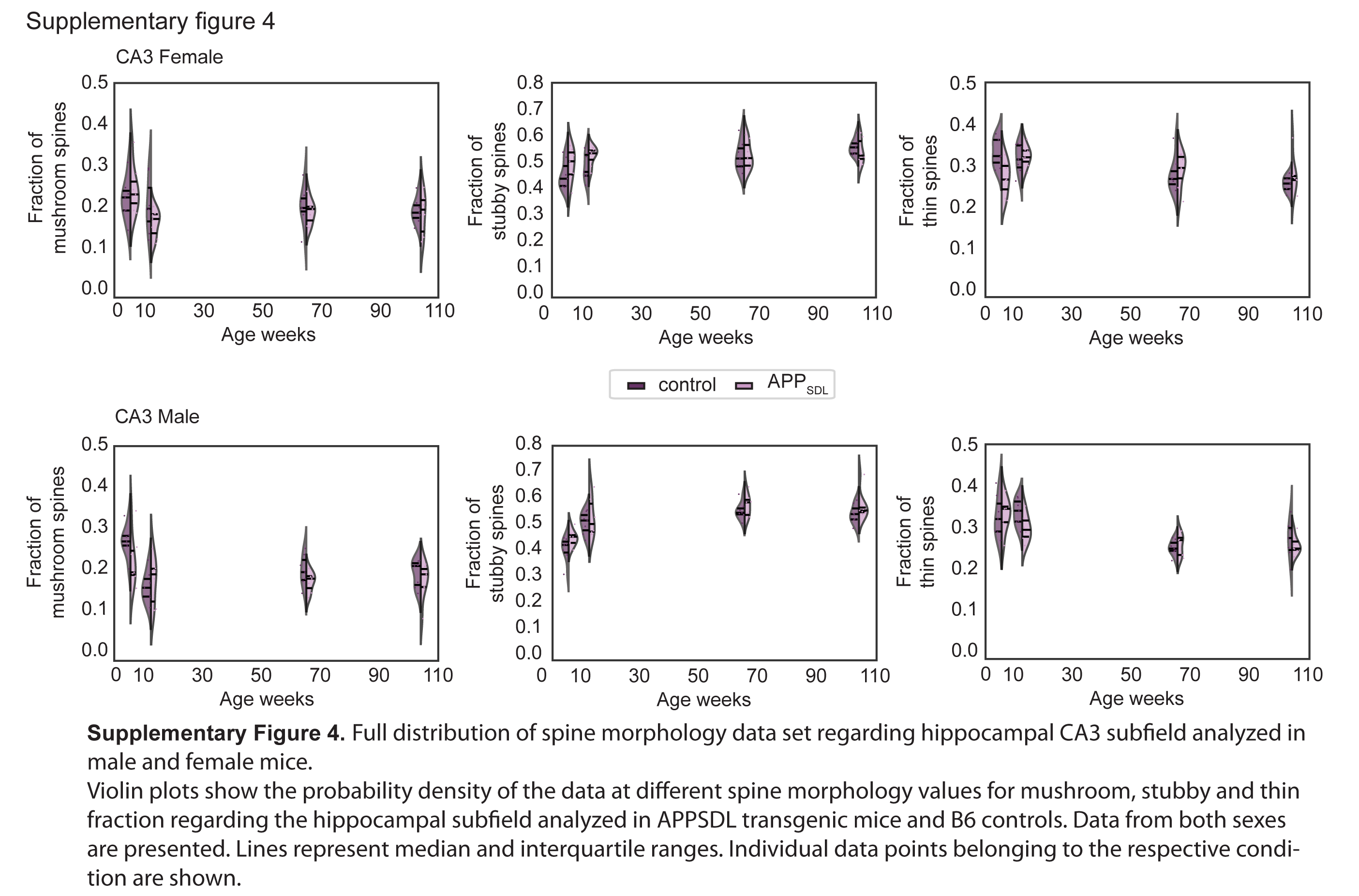

Supplement: Supplementary file 5 [file Image_4.TIF]

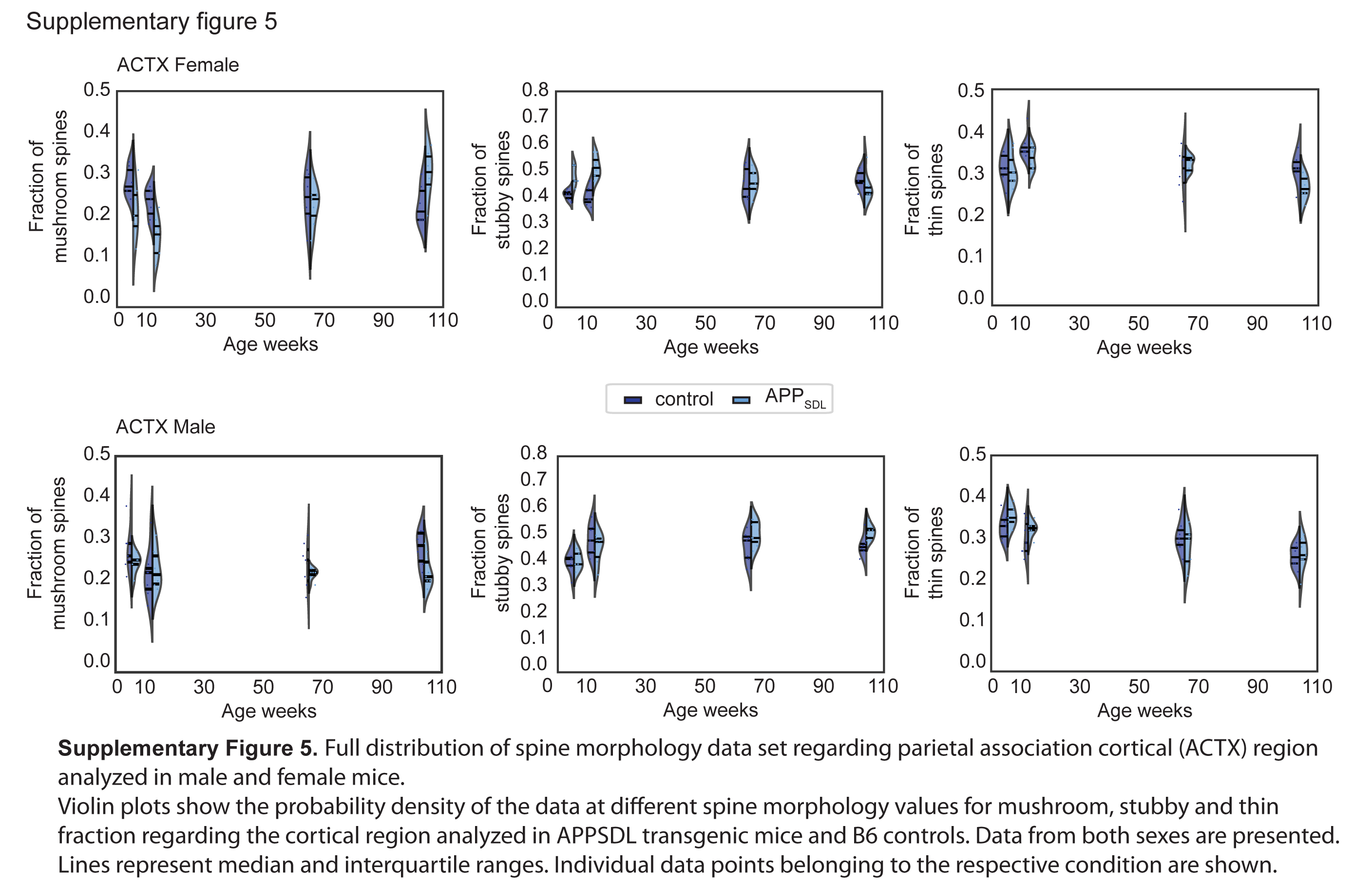

Supplement: Supplementary file 6 [file Image_5.TIF]

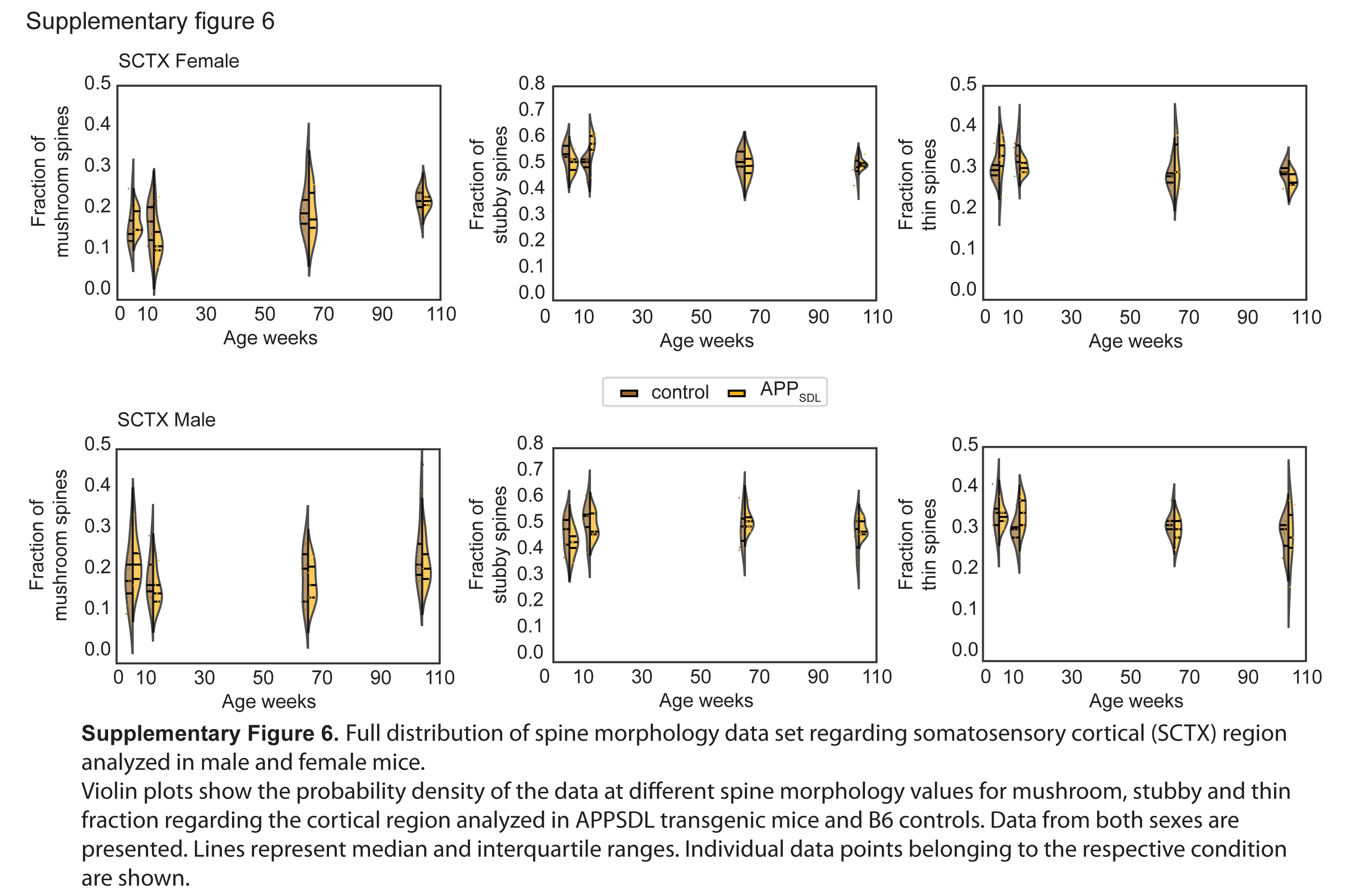

Supplement: Supplementary file 7 [file Image_6.TIF]
